# Diversity and Evolution of Myxobacterial Type IV Pilus Systems

**DOI:** 10.3389/fmicb.2018.01630

**Published:** 2018-07-19

**Authors:** Gaurav Sharma, Lori L. Burrows, Mitchell Singer

**Affiliations:** ^1^Department of Microbiology and Molecular Genetics, University of California, Davis, Davis, CA, United States; ^2^Department of Biochemistry and Biomedical Sciences and the Michael G. DeGroote Institute for Infectious Diseases Research, McMaster University, Hamilton, ON, Canada

**Keywords:** phylogeny, evolution, motility, pilus, Deltaproteobacteria, genome-organization, taxonomy, soil microbe

## Abstract

Type IV pili (T4P) are surface-exposed protein fibers that play key roles in the bacterial life cycle via surface attachment/adhesion, biofilm formation, motility, and development. The order Myxococcales (myxobacteria) are members of the class Deltaproteobacteria and known for their large genome size and complex social behaviors, including gliding motility, fruiting body formation, biofilm production, and prey hunting. *Myxococcus xanthus*, the best-characterized member of the order, relies on the appropriate expression of 17 type IVa (T4aP) genes organized in a single cluster plus additional genes (distributed throughout the genome) for social motility and development. Here, we compared T4aP genes organization within the myxobacteria to understand their evolutionary origins and diversity. We found that T4aP genes are organized as large clusters in suborder Cystobacterineae, whereas in other two suborders Sorangiineae and Nannocystineae, these genes are dispersed throughout the genome. Based on the genomic organization, the phylogeny of conserved proteins, and synteny studies among 28 myxobacterial and 66 Proteobacterial genomes, we propose an evolutionary model for the origin of myxobacterial T4aP genes independently from other orders in class Deltaproteobacteria. Considering a major role for T4P, this study further proposes the origins and evolution of social motility in myxobacteria and provides a foundation for understanding how complex-behavioral traits, such as gliding motility, multicellular development, etc., might have evolved in this diverse group of complex organisms.

## Introduction

Surface adhesion and motility are important bacterial traits. Surface filaments such as pili can promote attachment while allowing for both twitching and social motility in a wide range of species (Wall and Kaiser, 1999; Merz et al., 2000; Sun et al., 2000; Mattick, 2002; Mauriello and Zusman, 2007; Nakane, 2015; Nan and Zusman, 2016). Type IV pili (T4P) are found in archaea (Makarova et al., 2016), Gram-negative (e.g., Proteobacteria and Cyanobacteria), and Gram-positive bacteria (Firmicutes, Actinobacteria, and Deinococcus-Thermus), making them one of the most diverse pilus systems (Gurung et al., 2016). Besides adhesion and motility, T4P play important roles in a variety of other cellular functions, including DNA transfer, phage binding, microcolony/biofilm formation, cell aggregation, protein secretion, and predation (Aas et al., 2002; Klausen et al., 2003; Hager et al., 2006; Evans et al., 2007; Bahar et al., 2009; Metzger and Blokesch, 2014; Bordeleau et al., 2015; Taktikos et al., 2015; Maldarelli et al., 2016). One unique feature of T4P is their ability to rapidly and repeatedly extend and retract due to ATP-dependent polymerization and depolymerization of pilin subunits (Mattick, 2002; Burrows, 2012; Chang et al., 2016).

The T4P apparatus is classified as T4aP or T4bP based on differences in the pilins and assembly system components. T4aP aid in dispersal across various surfaces via flagella-independent twitching motility, whereas T4bP are usually involved in adherence and aggregation (Ayers et al., 2010). The T4aP machinery is composed of more than 20 different proteins, and its function is controlled by a number of regulatory components in response to poorly defined environmental cues (Leighton et al., 2015). The nomenclature of T4aP proteins is not well harmonized, but herein we use *Pseudomonas aeruginosa* and *Myxococcus xanthus* nomenclature, which is identical for most components. Because *pilA*, *pilB*, *pilC*, *pilD*, *pilM*, *pilN*, *pilO*, *pilP*, *pilQ*, and *pilT* encode the main components of the T4aP machinery, they are referred to as core genes (Nudleman and Kaiser, 2004), whereas non-core genes (e.g., *pilE*, *pilF*, *pilG*, *pilH*, *pilI*, *pilJ*, *pilK*, *pilR*, *pilS*, *pilU*, *pilV*, *pilW*, *pilX*, *pilY1*, *pilZ*, *fimU*) are less conserved. Among these, *pilA* encodes the major pilin whereas *fimU*, *pilV*, *pilW*, *pilX*, and *pilE* encode minor (low abundance) pilins that form a putative subcomplex with adhesin PilY1 (Nguyen et al., 2015). Here our focus is on the structural components of the T4P system, which are either present in a cluster or randomly scattered in the genome.

The T4aP machine is a large multi-protein complex that spans the entire cell envelope in Gram-negative bacteria. It consists of four sub-complexes: the pilus, the outer membrane subcomplex, the motor, and the alignment subcomplex. The pilus filament is composed mainly of PilA subunits, plus the minor pilin-PilY1 subcomplex. The pilus filament can be extended and retracted by the addition and removal of PilA subunits at the inner membrane via ATP-dependent polymerization and depolymerization activities of PilB and PilT (Mancl et al., 2016; McCallum et al., 2017). PilA proteins have a conserved N-terminal hydrophobic α-helix, which forms the core of the pilus filament, and hydrophilic C-terminal domain, which forms the outer surface and terminates in a characteristic disulfide-bonded loop (DSL) or D-region (Craig et al., 2006; Harvey et al., 2009; Hospenthal et al., 2017). The DSL has been reported to mediate attachment of pili to biotic/abiotic surfaces (Giltner et al., 2006). The outer membrane complex is composed of PilQ [a 14-membered gated pore that allows the pilus to cross the outer membrane (Koo et al., 2016)] and PilF (the pilotin lipoprotein that assists in correct localization and oligomerization of PilQ) (Burrows, 2012; Leighton et al., 2015). In *M. xanthus* and *Neisseria gonorrhoeae*, this complex also includes TsaP, which helps to anchor PilQ to the peptidoglycan (Siewering et al., 2014; Chang et al., 2016). The motor subcomplex is minimally composed of PilB, PilC, and PilT (Wall and Kaiser, 1999; Nudleman et al., 2006), although some species have one or more additional PilT-like proteins (Whitchurch and Mattick, 1994; Chiang et al., 2008), e.g., four PilT proteins in *M. xanthus* (Clausen et al., 2009). The PilB ATPase is involved in pilin polymerization (assembly) and PilT in pilin depolymerization (disassembly) (Chiang et al., 2008; Jakovljevic et al., 2008; Bischof et al., 2016; McCallum et al., 2017). *pilD* encodes a bi-functional integral membrane enzyme that removes the signal peptide from the pilin subunits and methylates the N-terminus of processed pilins (Strom et al., 1994). The alignment subcomplex, composed of PilMNOP, links the outer and motor subcomplexes, and regulates pilus extension-retraction dynamics in an unknown manner (Leighton et al., 2016).

The co-transcribed *pilMNOPQ* genes are characteristic of most bacteria that express T4aP, while the remaining genes are spread throughout the genome as smaller, transcriptionally distinct units (Pelicic, 2008). One exception is *M. xanthus* DK1622 (Wall and Kaiser, 1999), where the *pil* cluster contains seventeen genes (Wall and Kaiser, 1999; Nudleman and Kaiser, 2004). *M. xanthus* DK1622 is an aerobic rod-shaped member of order Myxococcales that are well known for gliding motility (adventurous and social motility), social behaviors, developmental programs, large genomes, and complex regulatory networks (Velicer and Vos, 2009; Kaiser et al., 2010). Gliding motility in myxobacteria coordinately integrates two-distinct mechanisms; adventurous motility (A-motility) when the myxobacterial cells are alone and social motility (S-motility) when myxobacterial swarms move together (Mauriello and Zusman, 2007; Nakane, 2015). T4P are involved in social motility but not in A-motility (Mauriello et al., 2010; Nan et al., 2011). Although Myxococcales are usually aerobic and classified as Deltaproteobacteria, other species in that class are typically anaerobes and syntrophic, procuring energy from sulfate and sulfur-reduction, and ferric iron reduction (Sanford et al., 2016). Myxococcales is subdivided into three suborders: Cystobacterineae, Nannocystineae, and Sorangiineae (Reichenbach and Dworkin, 1992; Shimkets et al., 2006). Most research on myxobacterial physiology and genetics has been performed using *M. xanthus*, a Cystobacterineae member, and it is not clear whether the findings are broadly applicable (Wall and Kaiser, 1999; Sogaard-Andersen, 2004; Nudleman et al., 2006; Mauriello and Zusman, 2007; Wu et al., 2007; Kaiser, 2008; Kaiser et al., 2010). The three suborders are quite diverse, based on genomic and comparative physiological studies. Many of the genes involved in motility and fruiting body development in *M. xanthus* (Cystobacterineae) are absent in suborders Nannocystineae and Sorangiineae (Huntley et al., 2011; Arias Del Angel et al., 2017). Here we examined the diversity and organization of T4aP genes across all sequenced myxobacteria and compared their organization with those of other closely related Deltaproteobacteria to understand their evolution among the three suborders within order Myxococcales.

## Materials and Methods

### Data Resource for Comparative Analysis

Genome sequences of all available strains of order Myxococcales and representative members of outgroups for this study (α-Proteobacteria, β-Proteobacteria, δ-Proteobacteria, γ-Proteobacteria, ε-Proteobacteria, Firmicutes, and Actinobacteria) were downloaded from the NCBI FTP site^1^ (**Supplementary Table S1**). No specific criteria were used to select the representative members. For representative members of order Myxococcales, we downloaded *Myxococcus hansupus* (*Mh*: CP012109) (Sharma et al., 2016b), *M. xanthus DK1622* (*Mx*DK1622: NC_008095.1) (Goldman et al., 2006), *M. fulvus HW-1* (*Mf*: NC_015711.1) (Li et al., 2011), *M. fulvus* 124B02 (MfB; CP006003) (Chen et al., 2016), *M. stipitatus*^T^ (*Ms*: NC_020126.1) (Huntley et al., 2013), *M. xanthus DZ2* (*Mx*DZ2: AKYI00000000) (Muller et al., 2013a), *M. xanthus DZF1* (*Mx*DZF1: AOBT00000000) (Muller et al., 2013b), *Anaeromyxobacter dehalogenans* 2CP-1 (*Ad1*: NC_011891.1), *Archangium gephyra* DSM 2261^T^ (*Ag*: CP011509.1) (Sharma and Subramanian, 2017), *Cystobacter fuscus* DSM 2262^T^ (*Cyb*: ANAH00000000) (Sharma et al., 2018), *Cystobacter violaceus* Cb vi76 (*Cyvi*: JPMI000000000) (Stevens et al., 2014), *Hyalangium minutum* DSM 14724^T^ (*Hm*: JMCB00000000) (Sharma et al., 2018), *Stigmatella aurantiaca* DW4/3-1 (*Sa*: NC_014623.1) (Huntley et al., 2011), *Corallococcus coralloides* DSM 2259^T^ (*Cc*: NC_017030.1) (Huntley et al., 2012), *Haliangium ochraceum* SMP-2 DSM 14365^T^ (*Ho*: NC_013440.1) (Ivanova et al., 2010), *Enhygromyxa salina* DSM 15201 (*Es*: JMCC00000000), *Plesiocystis pacifica* SIR-1 (*Pp*: ABCS00000000), *Chondromyces apiculatus* DSM 436 (*Cap*: ASRX00000000) (Sharma et al., 2018), *Chondromyces crocatus* Cm c5 (Ccmc: CP012159.1) (Zaburannyi et al., 2016), *Sorangium cellulosum* So0157-2 (Han et al., 2013) (*So*0157: NC_021658.1), *S. cellulosum* Soce56 (Schneiker et al., 2007) (*Soce*56: NC_010162.1), *Labilithrix luteola* DSM 27648^T^ (*Ll*: CP012333.1), *Vulgatibacter incomptus* DSM 27710^T^ (*Vui*: CP012332.1), and *Sandaracinus amylolyticus* DSM 53668^T^ (*Samy*: CP011125.1) (Sharma et al., 2016a). Despite their origin from the same strain, we have used all three *M. xanthus* strains DK1622, DZ2, and DZF1 in this study (Dey et al., 2016). We performed gene prediction and functional annotation with RAST (Aziz et al., 2008).

For all the organisms under study, 16S rDNA sequences were extracted from the NCBI FTP site (see footnote 1). 16S rDNA sequences were aligned using MUSCLE in MEGA v7.0 (Tamura et al., 2013) and further used to generate a maximum likelihood (ML) phylogeny using the JTT matrix model with 100 bootstrap values. The obtained ML tree was visualized in iTOL (Letunic and Bork, 2011), and the T4aP genes distribution was mapped onto the tree.

### Identification of T4aP Genes, Architecture, Synteny, and Phylogeny

T4aP nomenclature in *M. xanthus* matches that used for *P. aeruginosa*. Based on previous literature, known PFAM domains, and sequence similarity, T4aP genes and predicted proteins were identified and extracted from *M. xanthus* DK1622 and *P. aeruginosa* PAO1 genomes. All genomes were scanned against the Pfam-A v29.0 database (Finn et al., 2014) with an *E*-value threshold of 0.00001 using hmmscan from the HMMER suite^2^ (Eddy, 2011) and further parsed using hmmscan-parser.sh to identify functional T4aP domains. Along with Pfam domain searches, we used T4aP protein sequences to perform Basic Local Alignment Search Tool (BLASTp/tBLASTn) searches (Altschul et al., 1997) against the predicted proteome/genome of each organism. Sequence hits below to the *E*-value of 0.00001, 35% query coverage and 35% similarity were not considered as a potential functional T4P protein. Based on the genomic location of genes within a respective chromosome or contig of a draft assembly, T4aP clusters were identified. All myxobacterial PilA proteins were aligned to *P. aeruginosa* PilA homolog structure (1OQW Chain-A) using Promals3D web server (Pei et al., 2008) followed by color coding using CHROMA (Goodstadt and Ponting, 2001). Myxobacterial PilA and PilT proteins were subjected to BLASTp (Camacho et al., 2009) against the protein sequences of studied organisms (*E*-value cutoff of 0.00001; minimum query coverage of 35% and minimum percent similarity of 35%). The N-terminal segment of the PilA subunit and the PilT protein are among the most conserved sequences characterizing the T4aP machinery. Moreover, we have used PilT as a phylogenetic marker to differentiate between T4P and Type 2 secretion system (T2S). All hits were checked for their annotation and domains, and protein sequences were aligned using MUSCLE program in MEGA v7.0 (Tamura et al., 2013). For the PilA phylogeny, only the highly conserved N-terminal alignment was used (the C-termini are divergent) whereas the complete PilT sequence was used for the PilT phylogeny. The alignment was used to generate a ML phylogeny using the JTT matrix model with 100 bootstrap values. NCBI BLAST+ (v 2.2.28+) and non-redundant protein (NR) database downloaded on 10-21-2016 was used throughout the study.

## Results

### Core T4aP Proteins Are Highly Conserved

**FIGURE 1:**
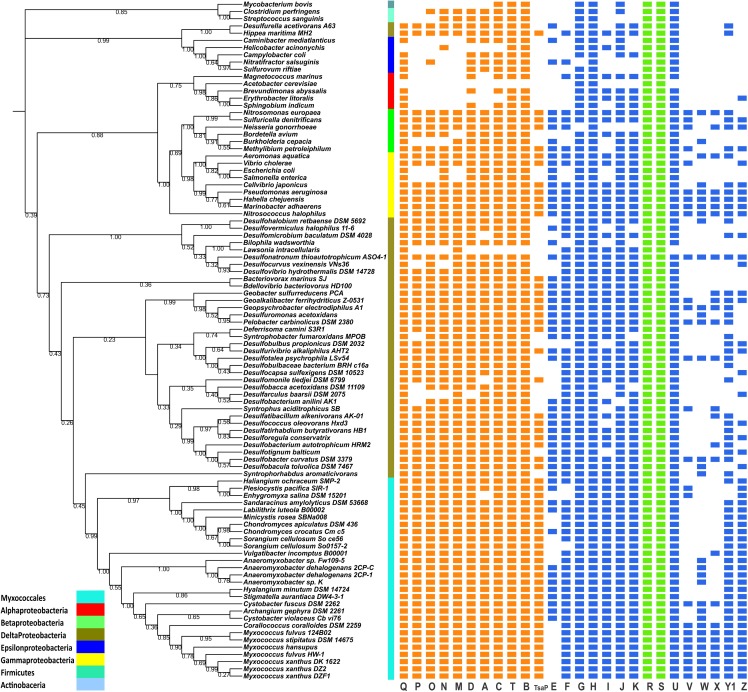
Distribution of T4aP genes among myxobacteria and outgroup organisms. The distribution of myxobacterial T4aP genes is mapped to a 16S rRNA-based phylogenetic tree using iTOL [https://itol.embl.de/]. Between the phylogenetic tree and gene distribution, taxonomy is mapped according to the color codes shown in the left corner. All genes are represented here with equal lengths and color codes: core T4P genes = orange; non-core T4P genes = blue; regulatory genes within T4P clusters (*pilR* and *pilS*) = green. Bootstrap values corresponding to the tree nodes are provided.

Of the predicted T4aP proteins identified in *P. aeruginosa* and *M. xanthus*, most were present among the 95 representative organisms used for this study. It is evident from the distribution (**Figure 1**) that the core proteins are well conserved (showing 50 to 100% identity) across most of the organisms studied, with the exception of α- and ε-proteobacteria as previously reported (Nudleman and Kaiser, 2004; Shelswell et al., 2005). Conservation of core T4aP genes is in support of the previous comparative genomic study by Pelicic (2008). In many organisms such as *Lawsonia intracellularis*, *Mycobacterium bovis*, and so on, homology searches could not identify several T4P proteins above the used cutoff values; therefore, we have reported them here as absent in the genome. In some places, a few core proteins were absent among representative organisms; their absence could be explained either by the draft nature of the genomic assembly or by an error in predicted start/stop codon’s gene calls in the automated assembly. Such changes may be due to authentic mutations or errors in sequencing. To distinguish between these two possibilities, further experimental investigations will be required. In genomes, such as *H. minutum*, the full T4aP cluster was present, but it was distributed within two contigs, i.e., contig 17 and 33. As other members of family Cystobacteraceae have a complete T4aP cluster at a single locus, we assume these contigs are likely to be contiguous in the *H. minutum* genome, although this hypothesis can be confirmed only when the assembly is completed.

Core genes *pilB*, *pilT*, *pilQ*, *pilC*, and *pilD* were highly conserved, and *pilMNOPQ* were present together in most of the species (**Figure 1**). We identified *tsaP* homologs within all myxobacteria and a few outgroup bacteria. In the *M. xanthus* DK1622 genome, two homologs of the regulatory genes *pilRS* were present within the single T4aP cluster (*pilR1S1* and *pilR2S2*). These genes encode a two-component histidine kinase-response regulator pair that controls pilus expression (Bretl et al., 2016). The Myxococcales are well known for having a large number of response regulators and histidine kinases compared to other bacteria (Whitworth and Cock, 2008; Whitworth, 2015; Sharma et al., 2018); we noted that *pilRS* homologs were present in all myxobacterial genomes and outgroups organisms (**Figure 1**). As T4P PilRS proteins are similar to other response regulators and histidine kinases present in the genome, we have shown them in **Figure 1** as a single occurrence depicting the presence of a response regulator and histidine kinase above used stringent cutoffs. Synteny and cluster organization studies were performed to identify both *pilR1S1* and *pilR2S2*, which are present in T4aP cluster (as in **Figure 3** and **Supplementary Figure S3**).

Compared to the core proteins, the rest of the T4aP proteins are less conserved. Although the literature suggests that *pilG*, *pilH*, and *pilI* are unique to the *Myxococcus* cluster (Wall and Kaiser, 1999), we found homology-based hits within almost all bacteria under study. Although supporting experimental data is not available, but it has been suggested that PilGHI proteins are involved in ABC transport and are sequentially similar to ABC transporters (Wall and Kaiser, 1999), which are present in multiple copies in genomes. We have considered sequence homology and related distance from the main cluster as one of the main identification for PilGHI. Three minor pilin clusters (two of *fimU-pilWVY1X* and one *pilW-Y1*) were often detected in the Myxococcaceae, whereas *M. stipitatus* and *C. coralloides* have a single cluster (**Supplementary Figure S3**). Comparative studies within seven *Myxococcus* species and in *Corallococcus* revealed that the *fimU-pilWVY1X* cluster was present in all but these two genomes, where all upstream and downstream genes were perfectly syntenic but the minor *pil* gene cluster, *fimU-pilWVY1X*, was deleted. Within the Cystobacteraceae members, only *C. fuscus* had a complete *fimU-pilWVY1X* cluster. Other members had *pilW*-x-*pilY1*, where *pilW* and *pilY1* were separated by one gene with distant sequence similarity to pilin-like proteins.

### Diversity of Myxobacterial PilA Proteins

Based on the Pfam domains (PF14245, PF00114, and PF07963) and homology searches against *M. xanthus* DK1622 and *P. aeruginosa* PAO1 PilA, we identified all PilA homologs in 95 bacterial genomes under study. As the T4P and type II secretion machinery share many similar functional domains (Ayers et al., 2010), we manually identified and removed all type II secretion machinery proteins from our datasets via mining their annotations and performing homology searches. Among different myxobacterial genomes, we found multiple *pilA* homologs ranging from one to seven (**Supplementary Figures S1, S3**). To identify conserved sites in PilA proteins, we structurally aligned the sequences of all order Myxococcales PilA proteins with the reference of *P. aeruginosa* strain PAK PilA (PDB id: 1OQW) (**Supplementary Figure S1**) and looked for conserved features of PilA proteins. Most of the predicted myxobacterial PilA proteins were well conserved in the first 60 residues of the N-terminus whereas the C-terminal domains had various deletions and insertions, making them more diverse. Within the Myxococcaceae, we identified two PilA homologs (∼30% identical to each other) in all three *M. xanthus* subspecies. The second PilA, which was encoded separately from the remaining T4aP components, has an extended but conserved N-terminal sequence, upstream of the predicted PilD cleavage site. Although the C-terminal Cys residues were absent in the second PilA, the adjacent residues were well conserved. Our study revealed that PilA homologs encoded within the T4aP cluster had conserved C-terminal Cys residues. Both *pilA*-encoded proteins in *S. aurantiaca* genome, which were present in a single T4aP cluster, had conserved Cys residues; it would be interesting to determine how the presence of two *pilA* genes affects T4aP assembly and function. Most of the family Cystobacteraceae PilA homologs, encoded separately from other T4aP components, had conserved Cys residues and conserved N-termini that were similar to that of PilA from Myxococcales T4aP clusters. One study in *Pseudomonas stutzeri* pointed out the presence of two *pilA* genes; one is the structural component whereas the second *pilA* ortholog (55% identical) antagonized genetic transformation (Graupner and Wackernagel, 2001). It would be fascinating to know whether these multiple orthologs of *pilA* in the various Myxococcales members have additional physiological functions as in *P. stutzeri*.

In most of the T4aP clusters, only a single *pilA* homolog was present, whereas *S. aurantiaca* encoded two tandem PilA proteins of 215 and 221 amino acids (similar in size to the 221-residue *M. xanthus* DK1622 PilA), sharing 70% similarity, suggesting a probable duplication event. It would be of interest to determine if one or both *pilA* are required for *S. aurantiaca* motility and development. Along with the *pilA* gene in T4aP cluster, family Cystobacteraceae members had additional *pilA* genes: two in *C. violaceus*, three in *A. gephyra*, five in *C. fuscus*, and seven each in *H. minutum* and *S. aurantiaca*. Most of these *pilA* genes were >60% similar to each other and formed a single phylogenetic clade, suggesting their origin through putative duplication. Within suborder Nannocystineae and Sorangiineae members, we found multiple *pilA* genes but they were not clustered with other T4aP genes. In the PilA proteins of the *Anaeromyxobacter* and *Vulgatibacter* T4aP clusters, only two Cys residues are present in the middle of C-terminal. A similar architecture was described for the *Dichelobacter nodosus* PilA homolog, FimA (Hartung et al., 2011). A similar arrangement of Cys residues was predicted for PilA homologs from suborder Nannocystineae and Sorangiineae members. PilA structure-based alignment suggested insertions of more than 50 amino acids within all myxobacterial PilA, although the insertions were at different locations in three suborders (**Supplementary Figure S1**).

### Phylogeny Based on T4aP Gene Organization

Using the conserved ∼60 residues N-termini alignment of PilA proteins, we generated a ML tree (**Supplementary Figure S2**) to identify the closest neighbors of the Myxococcales PilA homologs. Most of the T4aP cluster PilA homologs from suborder Cystobacterineae formed one clade (depicted by shaded text from the center of the tree), while those from the suborders Nannocystineae and Sorangiineae formed a separate clade. The suborder Cystobacterineae PilA proteins had close phylogenetic relationships with PilA homologs from the orders Bdellovibrionales and Desulfuromonadales (Deltaproteobacteria-I in **Supplementary Figure S2**), which is supported by their similar T4aP cluster organization as in **Figure 3**. This clade includes other myxobacterial PilA paralogs whose functions are still unknown; experimental studies will be necessary to define their putative role in myxobacteria. Both the *S. aurantiaca pilA* are together, suggesting they arose via duplication. T4aP organization among the outgroups revealed the presence of >2 *pilA* genes within *Pelobacter* and *Deferrisoma*. Similarity, in *Desulfuromonas acetoxidans*, three *pilA* genes were identified, separate from the main T4aP cluster. PilA protein-based phylogeny revealed that one PilA homolog from the T4aP cluster was present in the clade related to suborder Cystobacterineae whereas the second PilA was distinct from that clade, suggesting its higher similarity with other Delta proteobacterial PilA homologs. The second clade comprising the suborders Nannocystineae and Sorangiineae showed maximum relatedness with PilAs from other Deltaproteobacteria orders (Bradymonadales, Desulfarculales, Desulfobacterales, Desulfurellales, Desulfovibrionales, and Syntrophobacterales; Deltaproteobacteria-II in **Supplementary Figure S2**), suggesting a common ancestor. Moreover, the second clade also has a few suborder Cystobacterineae PilA homologs distributed in their genome, away from the main T4aP cluster. PilA homologs encoded within a T4aP cluster form a single clade, whereas other PilA homologs group together.

**FIGURE 2:**
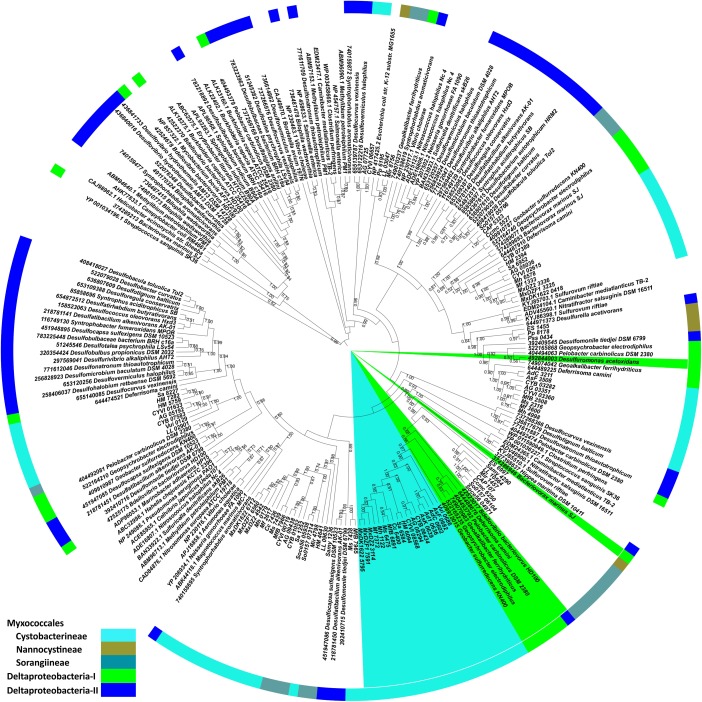
PilT protein-based maximum likelihood phylogeny. Myxobacterial *pilT* encoded proteins were subjected to BLASTp against the studied organisms. The top hits were extracted and aligned with myxobacterial homologs, which we further used for the construction of the phylogenetic tree. Myxobacterial and outgroup *pilT* homologs, which are a part of a T4aP cluster, are shaded throughout the full clade from the center. Bootstrap values (>50%) are depicted here adjacent to the tree nodes. In the outer circle, we mapped taxonomy according to color codes shown in the left corner. Deltaproteobacteria-I includes members of orders Bdellovibrionales and Desulfuromonadales whereas Deltaproteobacteria-II includes members of Bradymonadales, Desulfarculales, Desulfobacterales, Desulfurellales, Desulfovibrionales, and Syntrophobacterales.

Although most of the core proteins are well conserved, PilT was suggested to be a signature protein for T4aP, owing to its conservation across the diverse species *M. xanthus*, *P. aeruginosa*, and *N. gonorrhoeae* and its absence in the closely related type II secretion system (Wall and Kaiser, 1999). To confirm the predicted evolutionary path of myxobacterial T4aP, we generated a PilT protein-based ML tree (**Figure 2**) with 100 bootstrap values. This experiment confirmed that PilT homologs present in T4aP clusters of the suborder Cystobacterineae grouped together (as depicted by shaded text in **Figure 2**) along with the members of *Bacteriovorax*, *Deferrisoma*, *Geoalkalibacter*, *Geobacter*, *Geopsychrobacter*, and *Pelobacter* (Bdellovibrionales and Desulfuromonadales) (Deltaproteobacteria-I in **Figure 2**). Randomly distributed PilT myxobacterial homologs formed multiple clades with ancestors belonging to other class Deltaproteobacteria orders (Bradymonadales, Desulfarculales, Desulfobacterales, Desulfurellales, Desulfovibrionales, and Syntrophobacterales) (Deltaproteobacteria-II in **Figure 2**). These data further support our argument based on the PilA structural alignment (**Supplementary Figure S1**) and N-terminal PilA phylogeny (**Supplementary Figure S2**) that the T4aP clusters of suborder Cystobacterineae are distinct from those of suborders Nannocystineae and Sorangiineae, and likely evolved independently.

### Genomic Organization of T4aP Machinery Genes

**FIGURE 3:**
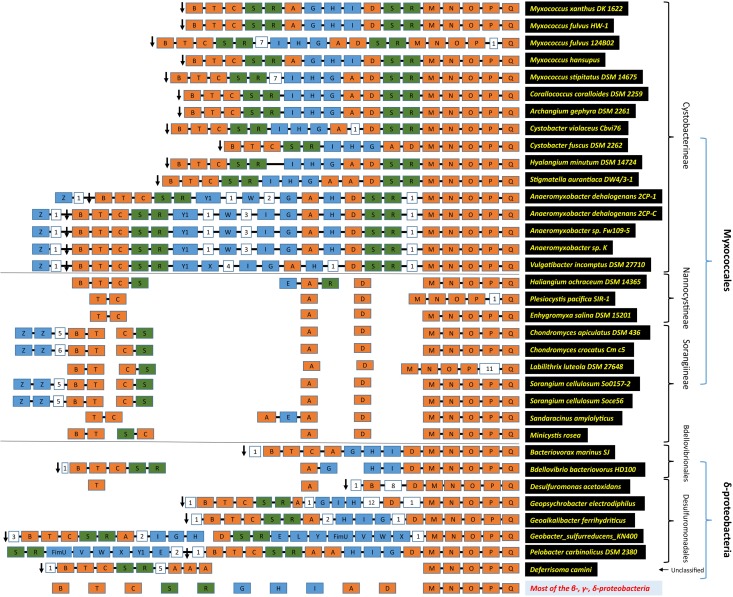
T4aP cluster architecture. The modular organization of the T4aP loci is depicted according to the direction of transcription as in *Mx*DK1622, going from left to right, using the color codes; core pili proteins as orange, rest of the pili genes as blue, and regulatory pili genes (*pilR* and *pilS*) as green. Digits in white boxes represent the number of intervening genes. The black arrow represents riboflavin kinase. Connecting black lines between boxes depict contiguous genes. Taxonomy and organism names are on the left.

Previously, it was suggested that homologs of the T4aP genes of *M. xanthus* and *A. dehalogenans* were present in a few members of class Deltaproteobacteria (Thomas et al., 2008). We compared the genomic organization of the Deltaproteobacteria T4aP genes in the order Myxococcales versus the non-Myxococcales members (**Figure 3**, **Supplementary Tables S2, S3** and **Supplementary Figure S3**). Within suborder Cystobacterineae, T4aP genomic organization was similar to that of *M. xanthus* DK1622 (Wall and Kaiser, 1999; Nudleman and Kaiser, 2004). Two minor pilin gene clusters of *fimU-pilWVY1X* and one cluster of *pilW*-x-*pilY1* were present apart from the main T4aP cluster in family Myxococcaceae, whereas only the *pilW*-x-*pilY1* cluster was present in family Cystobacteraceae. Among the family Cystobacteraceae members, *C. fuscus* lacked *pilR2S2*. It was reported (Bretl et al., 2016) that both *pilR1S1* and *pilR2S2* are necessary for T4aP assembly, and regulate the production of chains of outer membrane vesicles. These vesicles, also known as extracellular appendages, are generated in liquid culture of *M. xanthus* and are important for motility (Bretl and Kirby, 2016). Another member of the family Cystobacteraceae, *S. aurantiaca*, had tandem *pilA* genes, which is unusual. *Anaeromyxobacter* and *Vulgatibacter* had an additional group of genes, i.e., *pilW*-x-*pilY* in the T4aP cluster.

We examined the synteny of genes flanking the T4aP cluster as such analyses may provide additional information about its evolution. We extracted five genes up- and downstream of the T4aP cluster and examined their locations in all 95 organisms. Interestingly, a riboflavin kinase gene (*ribF*) was always present downstream of *pilB* (**Supplementary Table S2**) consistent with a previous report (Thomas et al., 2008). This bi-functional protein, RibF, converts riboflavin to FMN and FAD and has no known link with motility.

Within suborder Nannocystineae and Sorangiineae, only the *pilMNOPQ* genes were clustered, whereas other T4aP genes were dispersed throughout the genome (**Figure 3** and **Supplementary Figure S3**). *pilC* and *pilT* were together in some species, but other core pili genes such as *pilA*, *pilD*, *pilR*, *pilS*, and *tsaP* were always separate from the *pilMNOPQ* cluster. Although *pilB* and *ribF* are universally present in myxobacteria, they were genetically unlinked in suborders Nannocystineae and Sorangiineae.

In 66 outgroup organisms, only the *pilMNOPQ* genes were clustered, except in a few class Deltaproteobacteria organisms (*Bacteriovorax*, *Bdellovibrio*, *Desulfuromonas*, *Geoalkalibacter*, *Geobacter*, *Geopsychrobacter*, *Pelobacter*, and *Deferrisoma*). *Bacteriovorax* and *Bdellovibrio* are members of order Bdellovibrionales whereas *Desulfuromonas*, *Geoalkalibacter*, *Geobacter*, *Geopsychrobacter*, and *Pelobacter* belong to order Desulfuromonadales, and *Deferrisoma* belongs to unclassified Deltaproteobacteria. In these organisms, T4aP genes were arranged as in suborder Cystobacterineae, with some exceptions (**Figure 3** and **Supplementary Table S3**). Both sets of *pilRS* genes (i.e., *pilR1S1* and *pilR2S2*) were absent in *Bacteriovorax marinus* SJ. All T4aP genes were present in three groups in *Bdellovibrio bacteriovorus* HD100. A 14-kb insert containing 12 genes was found between *pilD* and *pilH* in *Geoalkalibacter ferrihydriticus* and *Geopsychrobacter electrodiphilus*. We identified two large clusters in *Geobacter sulfurreducens* KN400, one of which has an insertion containing minor pilin genes *pilELY1-fimU-pilVWX*. The T4aP cluster in *Pelobacter carbinolicus* DSM 2380 has two *pilA* genes and a minor pilin cluster of *fimU-pilVWXY1E*. In *Desulfuromonas acetoxidans*, *pilA* and *pilT* genes are unlinked from one another and the main T4aP cluster. *Deferrisoma camini* has three *pilA* homologs, and *pilDGHI* genes were present alone throughout the genome. All these species had a bi-functional riboflavin kinase gene downstream of *pilB* as in suborder Cystobacterineae (**Supplementary Table S3**). In rest of the orders of Deltaproteobacteria, the T4aP genes were separated from each other. Moreover, *pilB* and riboflavin kinase genes were located far apart on the chromosome.

## Discussion

**FIGURE 4:**
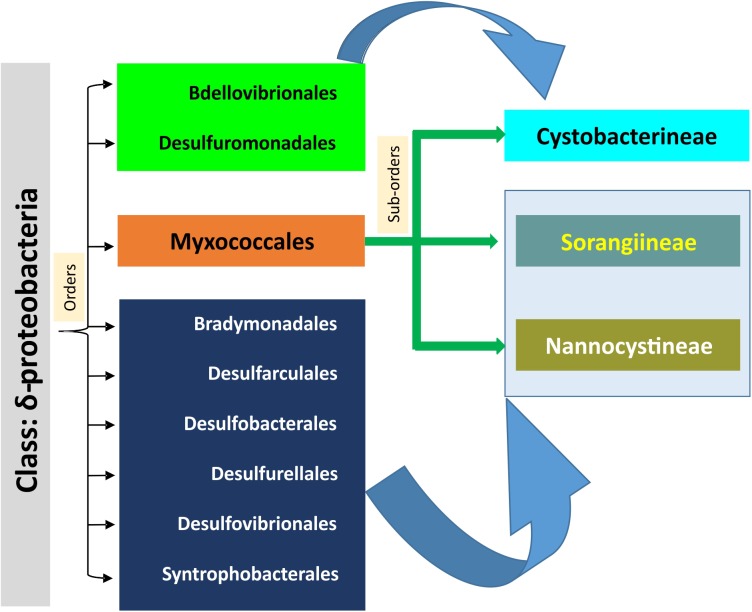
The final evolutionary model of myxobacterial T4aP cluster origin.

Social motility is an evolutionary feature distinct between myxobacteria and non-Myxococcales Deltaproteobacteria. How social motility evolved in myxobacteria has been a long-lasting question. Myxobacteria lack flagella but have T4P, which help their swarms to move on surfaces. Motility in myxobacteria is also regulated via multiple chemosensory systems (Sun et al., 2000; Zusman et al., 2007; Moine et al., 2014; Sharma et al., 2018) such as Frz (Che1) (Berleman et al., 2011), Che4, Che6 (Zusman et al., 2007), etc., extracellular polysaccharide (Black et al., 2006; Zhou and Nan, 2017) and lipopolysaccharide O-antigen (Vassallo et al., 2015), although the focus of this study is on T4P genes. As identified by large-scale comparative genomic analysis (Pelicic, 2008), most of the T4aP biogenesis genes are distributed throughout the genomes of Gram-negative bacteria, especially in Proteobacteria. Contradictory to this, the T4aP genes of *M. xanthus* and closely related members of suborder Cystobacterineae are clustered. Therefore, our focus here was to identify the potential origin of clustered T4aP gene organization in one branch of Proteobacteria, i.e., suborder Cystobacterineae of order Myxococcales. Our phylogenomic studies of myxobacterial T4aP gene diversity allowed us to propose an evolutionary model of their emergence (**Figure 4**). A comparable genomic organization of more than 15 T4aP genes in a single locus among the members of suborder Cystobacterineae, order Bdellovibrionales, and order Desulfuromonadales suggested that T4P systems among these organisms likely evolved from common ancestors. This hypothesis was further supported by the high relatedness of *pilA* and *pilT* genes from suborder Cystobacterineae T4aP clusters and their respective homologs from orders Bdellovibrionales and Desulfuromonadales (Deltaproteobacteria-I; **Figure 2** and **Supplementary Figure S2**). The presence of *ribF* downstream of *pilB*, only among the members of suborder Cystobacterineae and orders Bdellovibrionales and Desulfuromonadales further supported this proposition. The three suborders of order Myxococcales are quite diverse in physiology, genetic diversity, and synteny (Goldman et al., 2006; Thomas et al., 2008; Huntley et al., 2011). From this work, we propose that the suborder Cystobacterineae from order Myxococcales, and orders Bdellovibrionales and Desulfuromonadales of class Deltaproteobacteria, had common ancestors with the similar T4aP genomic organization, which further evolved in these groups of organisms. In addition, as suborders Nannocystineae and Sorangiineae from order Myxococcales and the remaining Deltaproteobacteria orders (Bradymonadales, Desulfarculales, Desulfobacterales, Desulfurellales, Desulfovibrionales, and Syntrophobacterales) (Deltaproteobacteria-II) have similar gene organization and share clades, we suggest that T4aP genes in these two Myxococcales suborders evolved separately from those in suborder Cystobacterineae. Our analysis suggests that the T4aP machinery within the three suborders of the order Myxococcales evolved in two different directions from the common ancestors of the class Deltaproteobacteria, resulting in two distinct genomic organizations (**Figure 4**). The results of this comparative study are expected to facilitate experimental characterization of unusual T4P systems in *Stigmatella*, *Anaeromyxobacter* etc., which might unearth new aspects of adventurous and social motility. Understanding the putative emergence of T4aP in myxobacteria will help us in further exploration of the origin of social motility and other characteristic myxobacterial features.

## Conclusion

Myxobacteria are aerobic Deltaproteobacteria with diverse characteristics such as gliding motility, sporulation, complex signal transduction, large genome, predation, and much more. Three suborders within order Myxococcales are quite diverse and distinct, both physiologically and genetically. Here we propose that myxobacterial T4aP system evolved in two different directions from the common ancestors of class Deltaproteobacteria. We present the comparative distribution of T4aP genes within 28 myxobacteria and 66 outgroup organisms from α-Proteobacteria, β-Proteobacteria, γ-Proteobacteria, δ-Proteobacteria, ε-Proteobacteria, Firmicutes, and Actinobacteria. Based on the similar genomic arrangement of T4aP genes, the relatedness of *pilA* and *pilT* homologs in respective phylogenies, and the presence of syntenic *ribF* downstream of *pilB*, we propose that the T4aP machinery within three suborders of order Myxococcales evolved independently from different orders of class Deltaproteobacteria. This independent evolution may have resulted in the two versions of T4aP gene organization now present within myxobacteria.

## Author Contributions

GS generated the idea, analyzed the distribution of T4aP in the organisms of interest, and performed the comparative and phylogenetic analysis. LB provided intellectual contributions for the analysis. MS provided funding and intellectual contributions. GS, LB, and MS wrote the manuscript.

## Conflict of Interest Statement

The authors declare that the research was conducted in the absence of any commercial or financial relationships that could be construed as a potential conflict of interest.

## Footnotes

1 ftp://ftp.ncbi.nlm.nih.gov/genomes/refseq/bacteria/

2 http://hmmer.janelia.org/

## Supplementary Material

FIGURE S1 Structure-based alignment of myxobacterial PilA homologs. Promals3D was used to align all myxobacterial PilA homologs against *Pseudomonas aeruginosa* PilA 1OQW_A chain, followed by alignment coloring by CHROMA (Goodstadt and Ponting, 2001). Green represents myxobacterial PilA proteins encoded within a T4aP cluster. Cys residues are shaded with magenta to show the conservation. Black shade with numbers indicates the number of collapsed amino acid residues in the alignment.

FIGURE S2 PilA N-terminal segment sequence-based maximum likelihood phylogeny. All myxobacterial *pilA* encoded proteins were subjected to BLASTp against the studied organisms. The top hits were extracted and aligned with myxobacterial PilA proteins. Out of 245 sequences, ones with the extended N-terminal were removed, and the conserved ∼60 AA from N-terminal of rest of the 209 sequences were used for building a maximum likelihood tree with RAxML. Myxobacterial and other *pilA* homologs encoded in T4aP clusters are shaded throughout the full clade from the center. Bootstrap values (>50%) are depicted adjacent to the tree nodes. In the outer circle, we mapped taxonomy according to color codes shown in the left corner. Deltaproteobacteria-I includes members of orders Bdellovibrionales and Desulfuromonadales whereas Deltaproteobacteria-II includes members of Bradymonadales, Desulfarculales, Desulfobacterales, Desulfurellales, Desulfovibrionales, and Syntrophobacterales.

FIGURE S3 Genomic organization of myxobacterial T4aP genes. The modular organization of all T4aP genes, present in a cluster (the cluster order is according to the direction of transcription, going from left to right as in *Mx*DK1622) or scattered in the genome, is depicted using the color codes; core pili proteins as orange, rest of the pili genes as blue, and regulatory pili genes (*pilR* and *pilS*) as green. Digits in white boxes represent the number of intervening genes. Connecting black lines between boxes depict contiguous genes.

TABLE S1 Organisms included in this study. The taxonomic classification, which includes class, order, sub-order, and family, is also provided for clarity. Color codes differentiate classes.

TABLE S2 Myxobacteria T4aP cluster genes and synteny.

TABLE S3 Order Bdellovibrionales and Desulfuromonadales T4aP cluster genes and synteny.
