# Supplementary figures and images for: Diversity and Evolution of Myxobacterial Type IV Pilus Systems
Source: Front Microbiol. 2018 Jul 19;9:1630. doi: 10.3389/fmicb.2018.01630 (PMC6060248; doi:10.3389/fmicb.2018.01630)

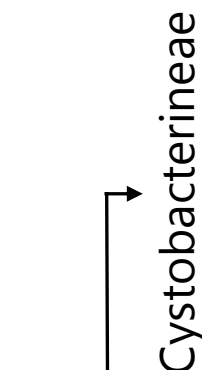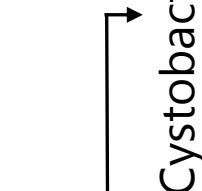

M.....taTLlElblVltllGlLAslAlssh.pb.pt+.tEsp..l.sl..s..s.....h..hsb...s.ba.Y.h

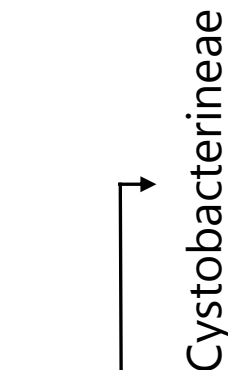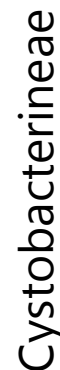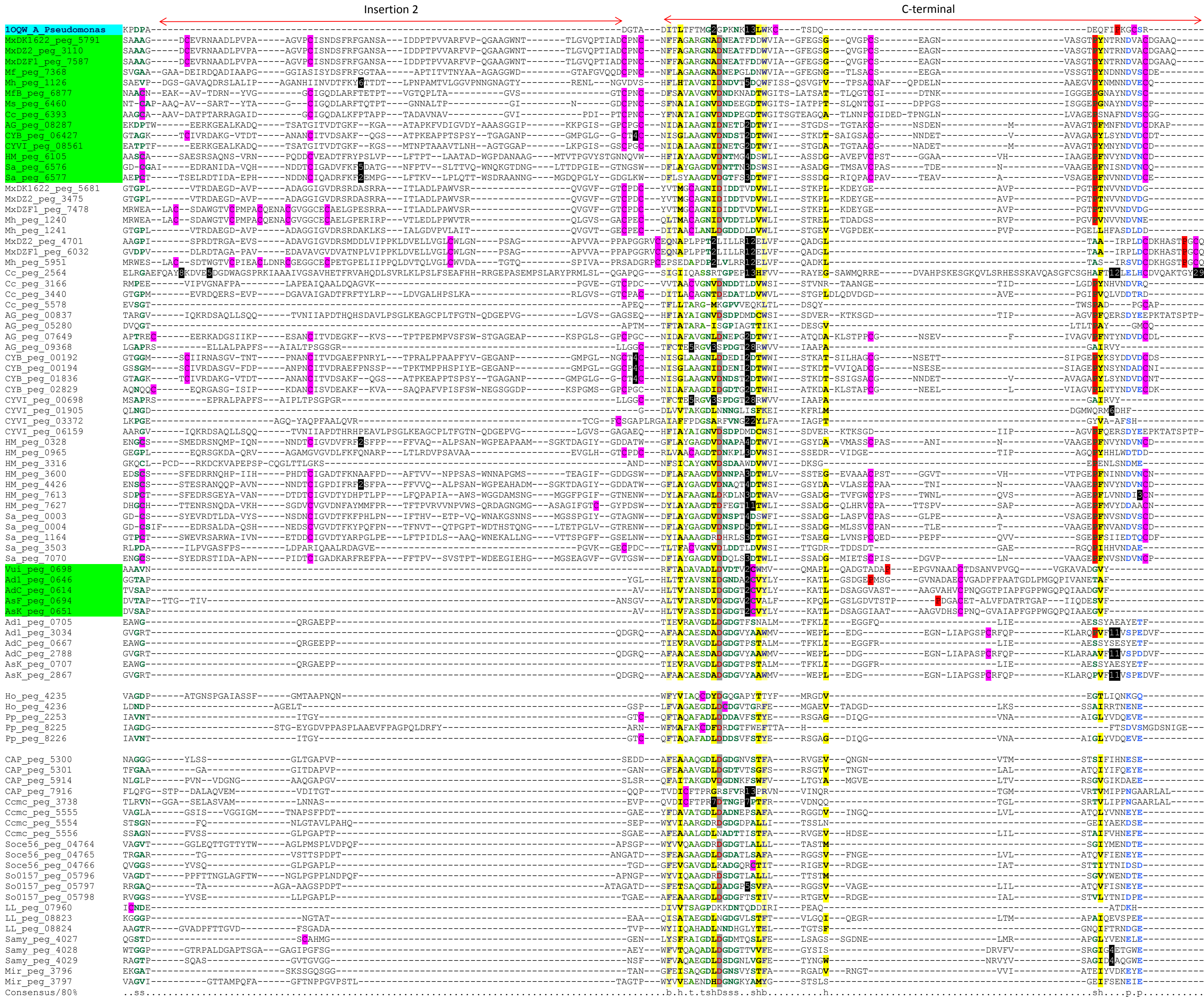

Supplement: FIGURE S1 — Structure-based alignment of myxobacterial PilA homologs. Promals3D was used to align all myxobacterial PilA homologs against Pseudomonas aeruginosa PilA 1OQW_A chain, followed by alignment coloring by CHROMA (Goodstadt and Ponting, 2001). Green represents myxobacterial PilA proteins encoded within a T4aP cluster. Cys residues are shaded with magenta to show the conservation. Black shade with numbers indicates the number of collapsed amino acid residues in the alignment. [file Presentation_1.PDF]

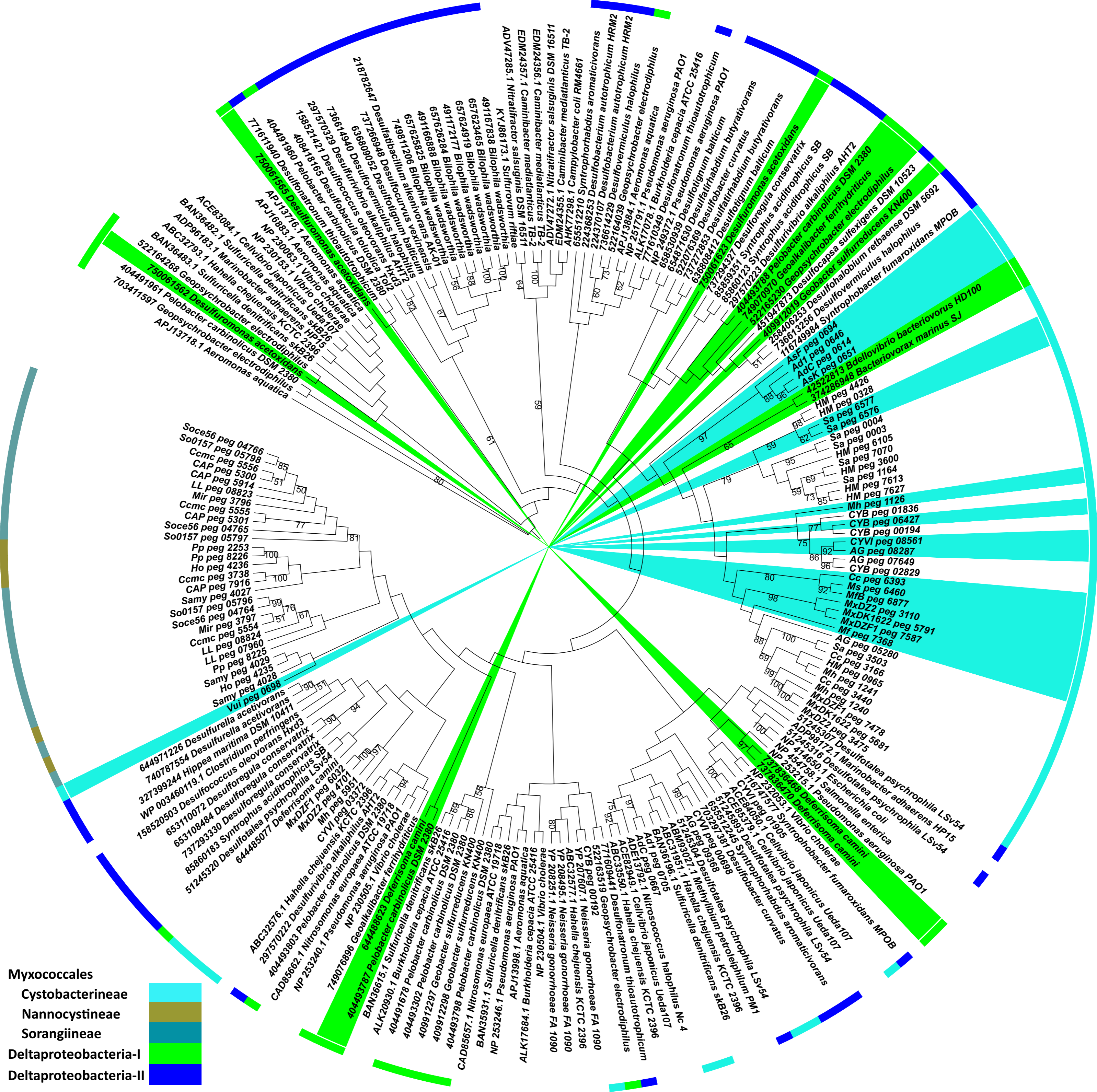

Supplement: FIGURE S3 — Genomic organization of myxobacterial T4aP genes. The modular organization of all T4aP genes, present in a cluster (the cluster order is according to the direction of transcription, going from left to right as in MxDK1622) or scattered in the genome, is depicted using the color codes; core pili proteins as orange, rest of the pili genes as blue, and regulatory pili genes (pilR and pilS) as green. Digits in white boxes represent the number of intervening genes. Connecting black lines between boxes depict contiguous genes. [file Data_Sheet_4.PDF]
