# Supplementary material for: Diversity and Evolution of Myxobacterial Type IV Pilus Systems
Source: Front Microbiol. 2018 Jul 19;9:1630. doi: 10.3389/fmicb.2018.01630 (PMC6060248; doi:10.3389/fmicb.2018.01630)
Supplement: FIGURE S2 — PilA N-terminal segment sequence-based maximum likelihood phylogeny. All myxobacterial pilA encoded proteins were subjected to BLASTp against the studied organisms. The top hits were extracted and aligned with myxobacterial PilA proteins. Out of 245 sequences, ones with the extended N-terminal were removed, and the conserved ∼60 AA from N-terminal of rest of the 209 sequences were used for building a maximum likelihood tree with RAxML. Myxobacterial and other pilA homologs encoded in T4aP clusters are shaded throughout the full clade from the center. Bootstrap values (>50%) are depicted adjacent to the tree nodes. In the outer circle, we mapped taxonomy according to color codes shown in the left corner. Deltaproteobacteria-I includes members of orders Bdellovibrionales and Desulfuromonadales whereas Deltaproteobacteria-II includes members of Bradymonadales, Desulfarculales, Desulfobacterales, Desulfurellales, Desulfovibrionales, and Syntrophobacterales. [file Presentation_2.PDF]

Suborder Cystobacterineae

***Myxococcus xanthus* DK 1622**

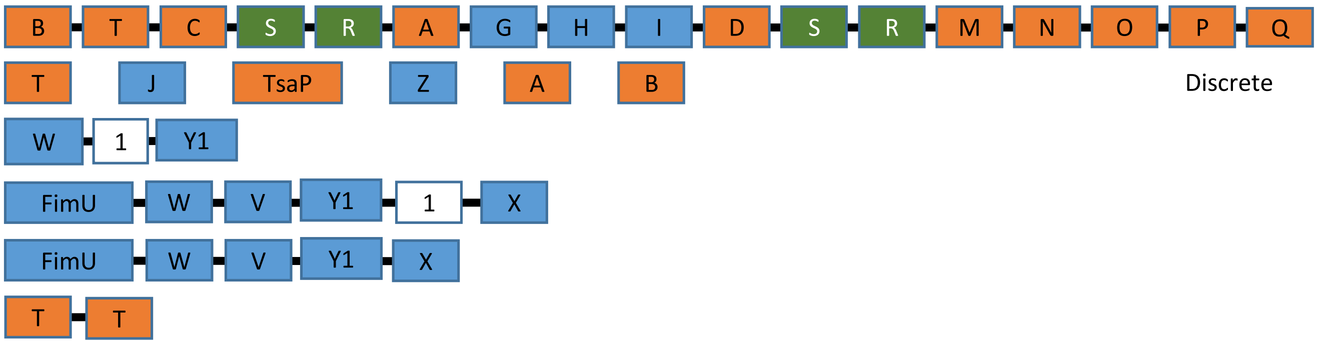

***Myxococcus xanthus* DZ2**

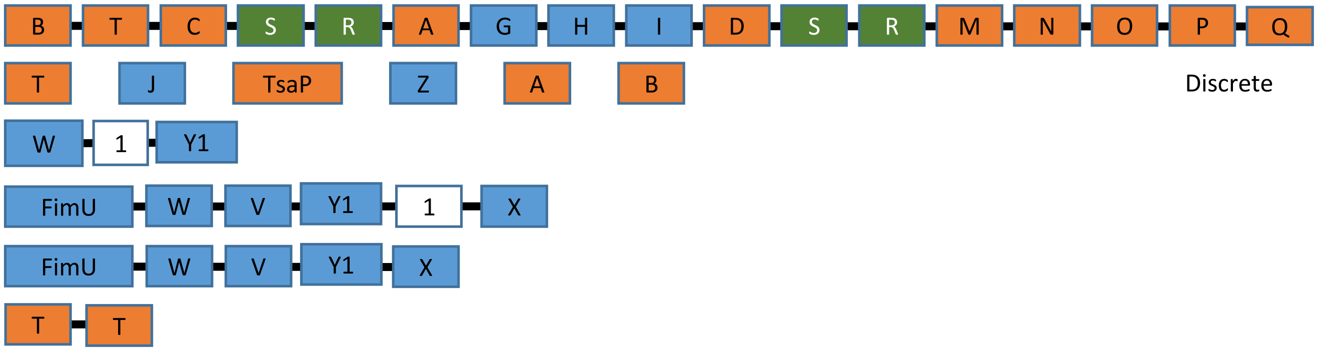

***Myxococcus xanthus* DZF1**

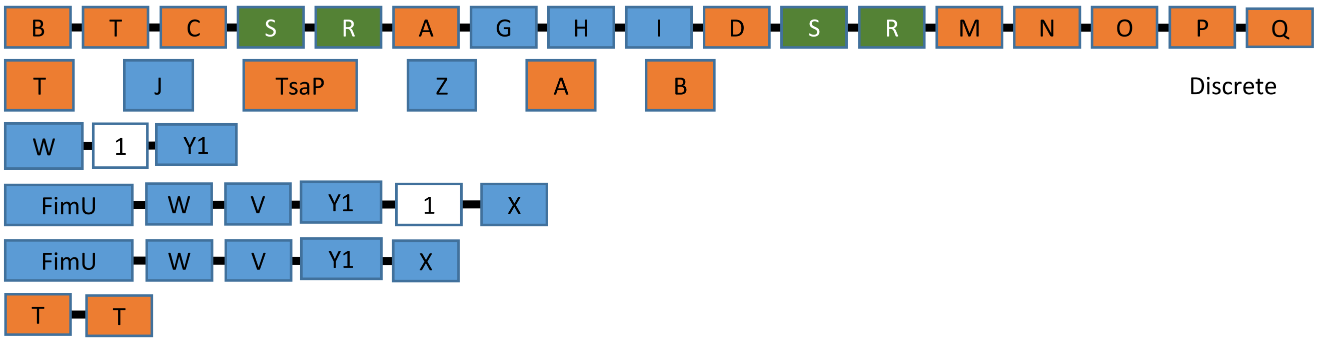

Supp. Figure 2

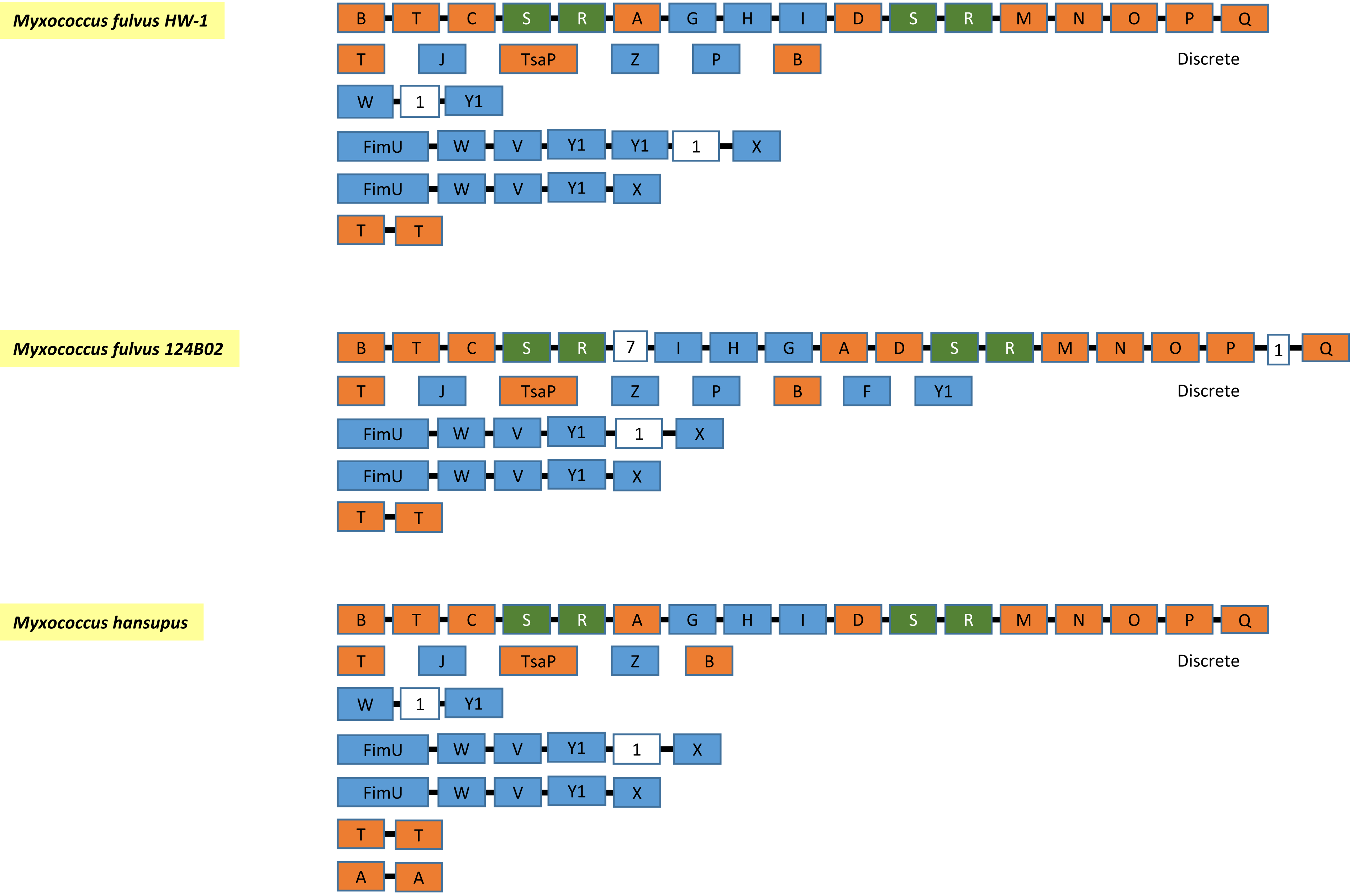

Supp. Figure 2

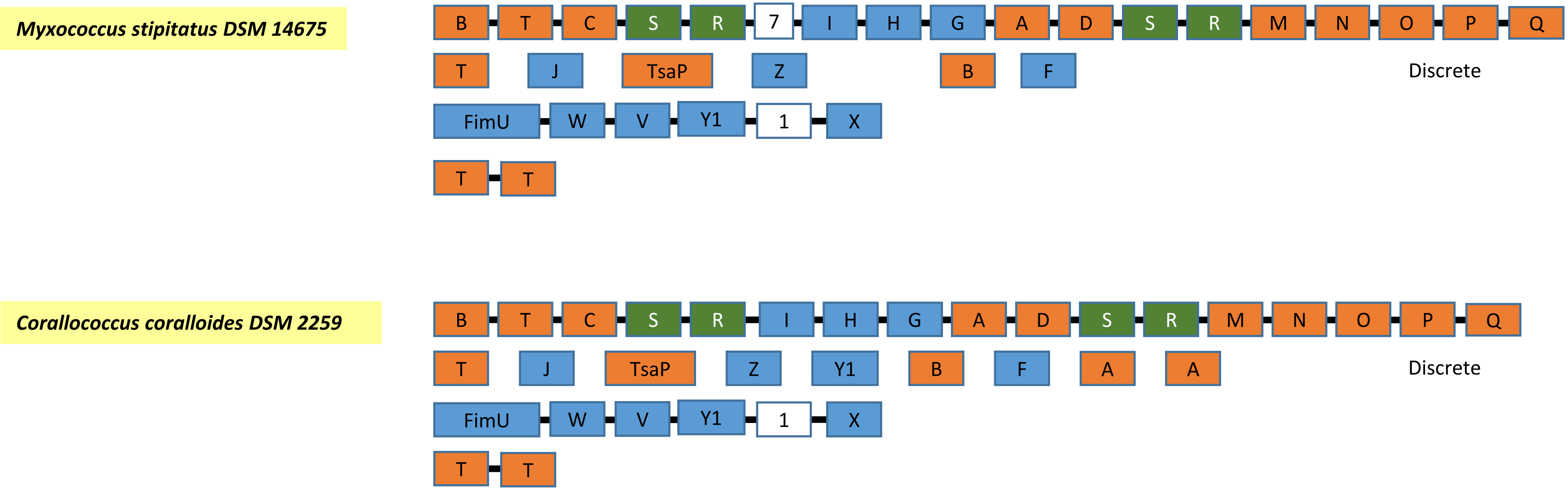

Supp. Figure 2

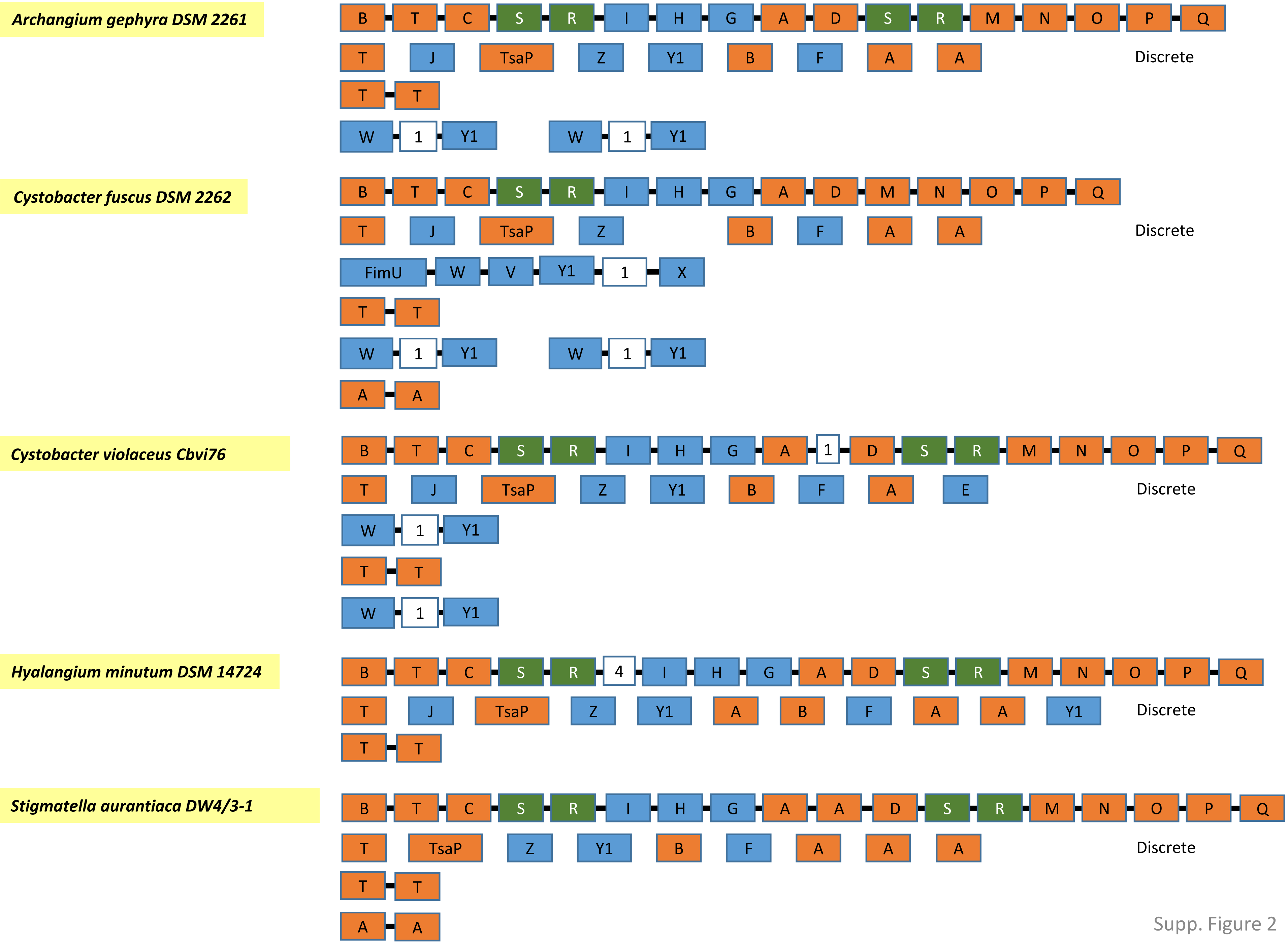

Supp. Figure 2

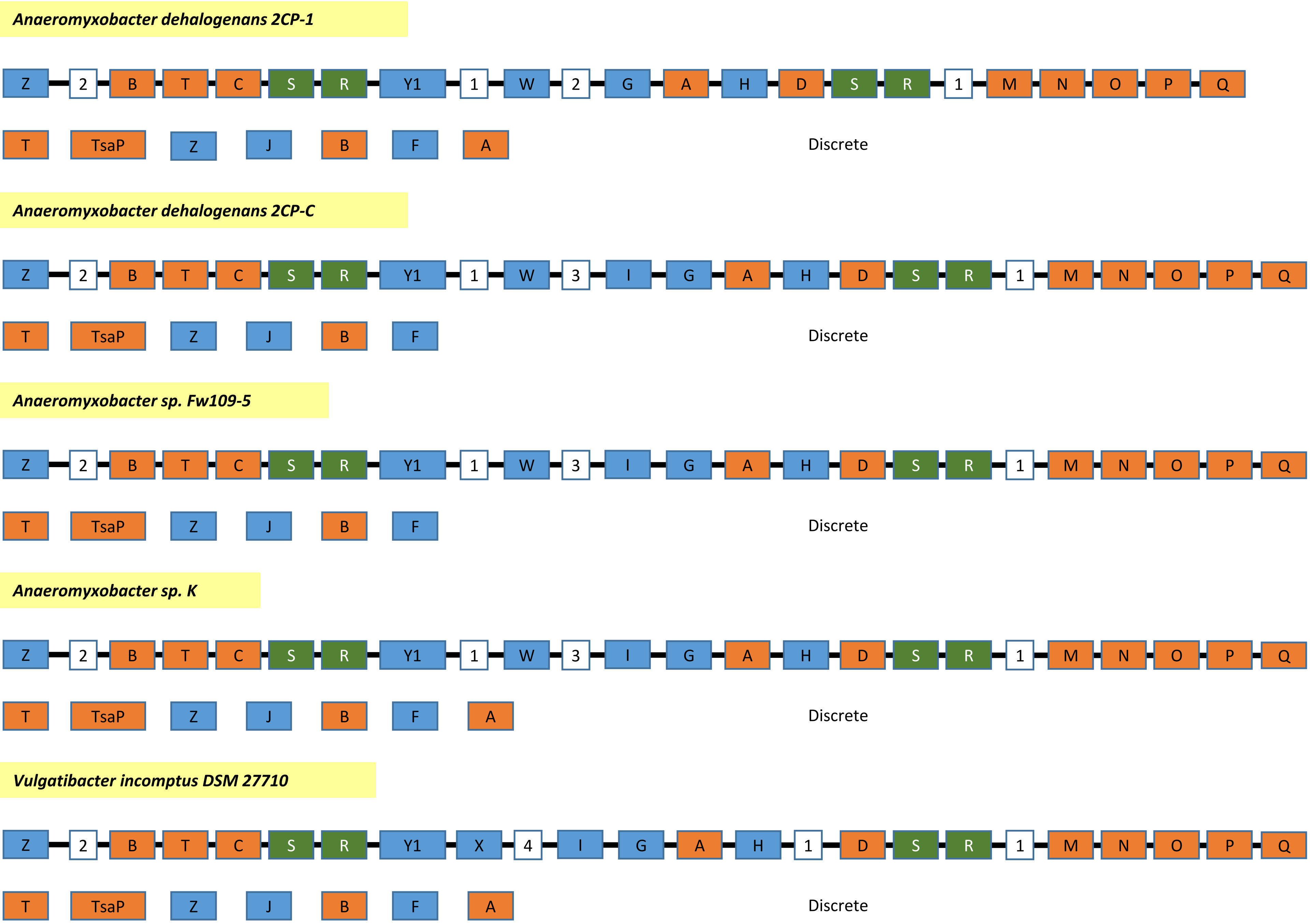

Supp. Figure 2

Suborder Nannocystineae

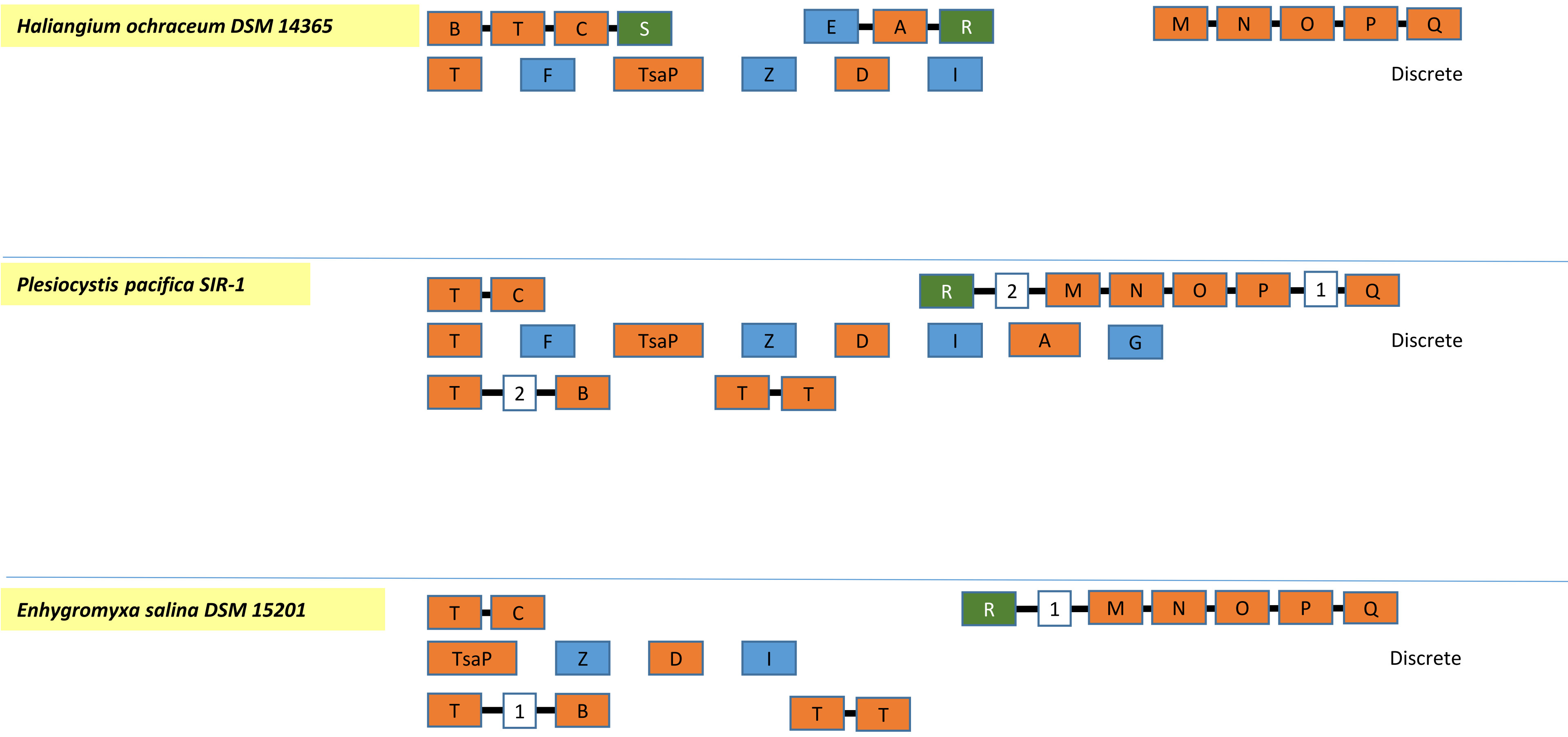

Supp. Figure 2

Suborder Sorangiineae

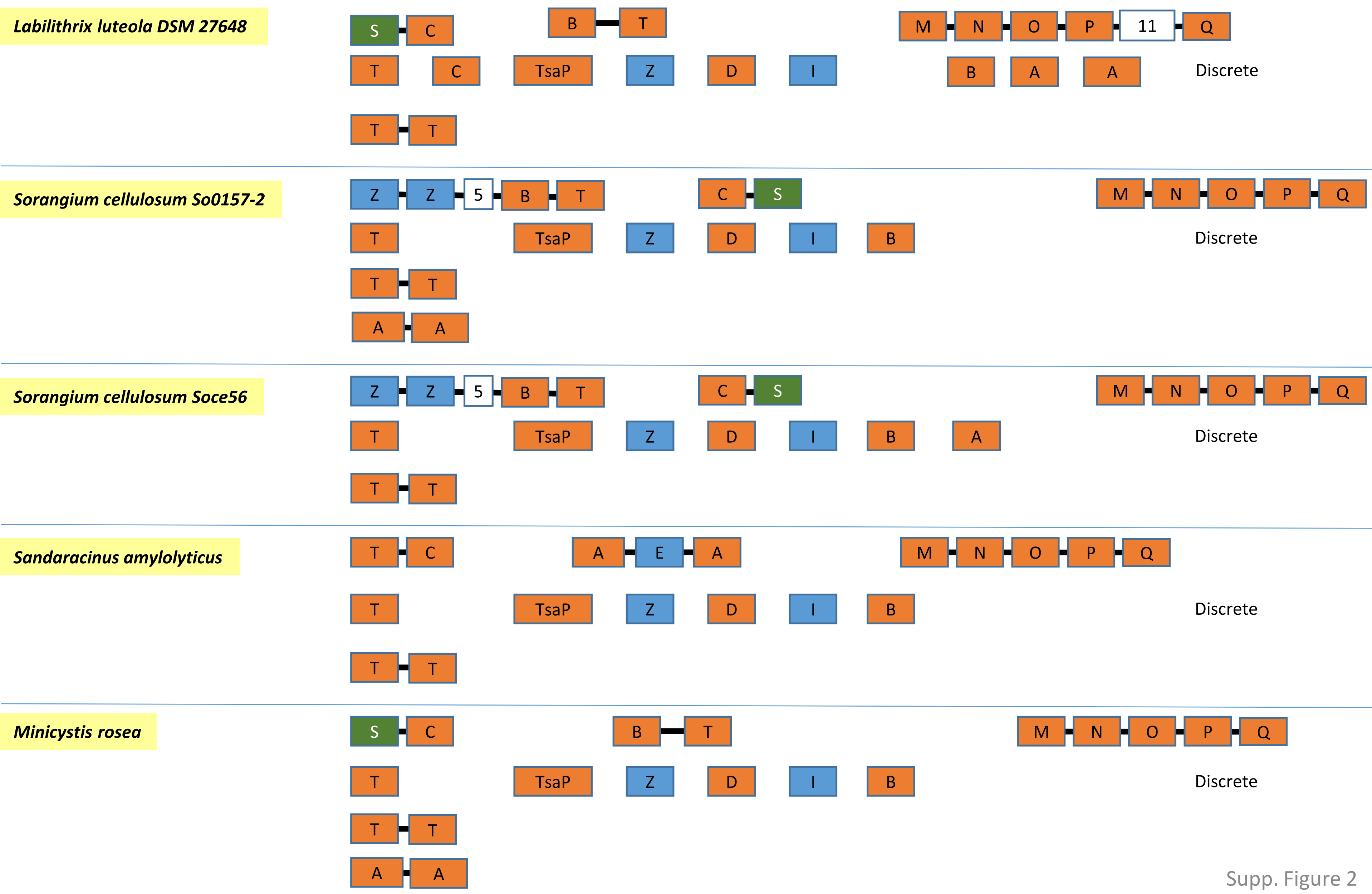

Supp. Figure 2

Chondromyces apiculatus DSM 436

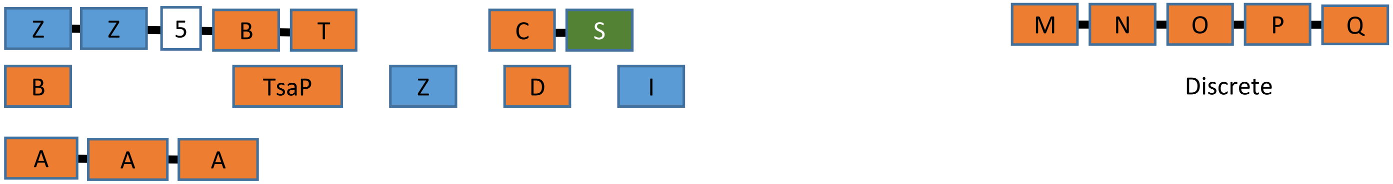

Chondromyces crocatus Cm c5

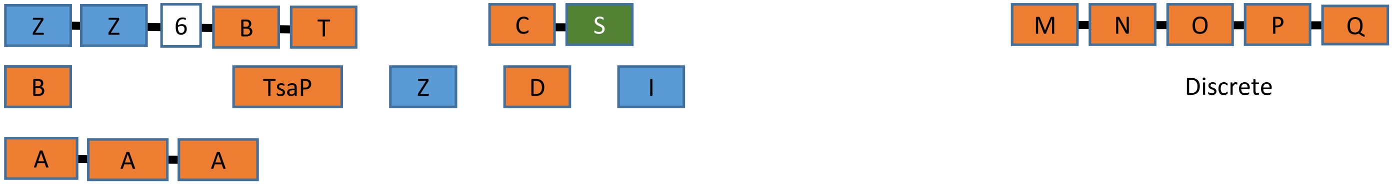

Supp. Figure 2
